# Editorial: Molecular Studies of Covid-19 Chemistry

**DOI:** 10.3389/fchem.2021.729142

**Published:** 2021-08-31

**Authors:** Domenica Capasso, Sonia Di Gaetano, Chandrabose Selvaraj, Emilia Pedone

**Affiliations:** ^1^ CIRPEB, University of Naples “Federico II”, Naples, Italy; ^2^ CESTEV, University of Naples “Federico II”, Naples, Italy; ^3^ Institute of Biostructures and Bioimaging, CNR, Naples, Italy; ^4^ Department of Bioinformatics, Alagappa University, Karaikudi, India

**Keywords:** repurposing drugs, SARS-CoV-2, inhibitor, host-virus intercation, design

This Research Topic on “*Molecular Studies of Covid-19 Chemistry*” aims to highlight current advances carried out at the molecular level, providing insight into the mechanism of infection of SARS-CoV-2. In particular, this Research Topic is devoted to studying unknown aspects of the SARS-CoV-2 infection process in order to identify new therapeutic targets and validate potential inhibitors. The published articles do so using multidisciplinary approaches that involve computational chemistry, chemical-physical, and biochemical methodologies. The researchers who contributed to this Research Topic present 18 themed articles that show the latest advances in newly identified targets against SARS-CoV-2.

Different authors have searched for novel leads from the available natural substances and clinically available drugs by targeting the SARS-CoV-2 Spike’s protein receptor binding domain (RBD), which remains by far the main target against the virus. Indeed, Carino et al. specifically target the druggable pockets located in the central β-sheet core in the RBD through virtual screening methods. This strategy suggests several triterpenoid/steroidal agents that block the protein-protein interactions between the RBD with human Angiotensin-Converting Enzyme 2 (ACE2). The findings of the computational screening are experimentally validated through ACE2/SARS-CoV-2 Spike Inhibitor Screening Assay Kit and from the analysis of Anti-SARS-CoV-2 IgG Antibodies. Overall, the authors have come up with strong potential triterpenoids, primary, secondary bile acids, and semi-synthetic derivatives for the RBD/ACE2 binding blockers reporting them as SARS-CoV-2 inhibitors. Gopinath et al. also identified new inhibitors for blocking the RBD-ACE2 interface. applying a unique protocol that combines mixed solvent MD simulations (MixMD) with high-throughput virtual screening (HTVS). MixMD are employed to identify the stable binding conformations of drug-like probes in the S-protein–ACE2 interface and those stable sites are applied for use in the molecular level screening. Among the compounds identified by MixMD, ZINC000002128789 showed a potential binding affinity to block the protein-protein RBD-ACE2 interface binding and prevent SARS-CoV-2 infection.

On the other hand, Tito et al. explored Plant-derived secondary metabolites as a source of potential inhibitors able to prevent and counteract the rapid spread of SARS-CoV-2 infections. By using *in vitro* approaches, the authors investigated the role of a pomegranate peel extract in attenuating the interaction between the SARS-CoV-2 Spike and ACE2, and on the activity of the virus 3CL protease. Although further studies are required to assess the efficacy of this extract *in vivo*, the results here reported open up new promising opportunities to employ natural extracts for the development of effective and innovative therapies in the fight against SARS-CoV-2.

Sakkiah et al. focus their research on interactions between the Spike and ACE2 building, a complex structure by homology modeling and by molecular dynamics simulations elucidating the interactions. In particular, the twenty interacting residues, responsible for binding, were characterized using Molecular Mechanics Poisson-Boltzmann Surface Area (MM/PBSA) and in silico alanine scanning.

One of the main targets against SARS-CoV-2 is the main viral protease (Mpro), a key protein involved in the replication process of the virus and therefore a relevant target for identifying high affinity molecules capable of selectively inhibiting it. This special issue contains different articles reporting in silico studies on potential inhibitors against Mpro. In particular, Yañez et al. used a computational model of Mpro built in complex with different synthetic ligands derived from coumarins and quinolones to identify new potential inhibitors. By an experimental approach based on molecular dynamics and molecular docking of the models, six compounds were selected as putative candidates. However, further biological studies are required to confirm the function of the selected compounds and enable the development of novel drugs that can be employed in SARS-CoV-2 therapy.

Mengist et al. have review the potential inhibitors targeting SARS-CoV-2 Mpro, providing insights into the Mpro mechanism and explaining its role as a drug target for inhibition of SARS-CoV-2. They review the active site structure, morphology for identifying the Structure-based Design of drugs. They discuss the design of the inhibitor based on their competitive binding to the active site, to identify the best candidates. Along with this, they examined all the reported compounds tested against the SARS-CoV-2 Mpro such as α-ketoamide inhibitors, peptide-based inhibitors, anilid-based inhibitors, screened molecules from the TCM (Traditional Chinese Medicine) database, plant compounds, and indole lactam-based inhibitors.

Selvaraj et al., reported conformational changes of SARS-CoV-2 Mpro in the long-range time scale event of 1 μs MD simulations. They have reported the crucial amino acids involved in the mechanism and protein stability through the 1 μs MD simulations and also reported a multiple conformation-based ensemble docking approach to screening potential SARS-CoV-2 Mpro inhibitors from the TCM database. Selvaraj et al. found the screened compounds from the TCM database that cause functional distortion of the oxyanion hole in the Mpro reaction mechanism, and the new leads show direct interactions with His41, Gly143, and Cys145. Through the induced fit docking, they found possible binding conformations, that have the ability to interact with residues and disturb the formation of the oxyanion hole, leading to its inhibition. Abel et al. have performed the combined structure and ligand based virtual screening methodology, which includes molecular fingerprints and molecular docking methods for identifying the SARS-CoV-2 Mpro inhibitors Super Natural II and TCM database. Mass evaluation of 80 docked complex was examined in detail, and among the four compounds tested for toxicity, cytochrome inhibition profiles and dynamically simulated for understanding its stability and interactions.

The papain-like protease (PlPro) is also considered another important drug target that plays the imperial role in viral maturation and although it is responsible for the essential mechanism, it is a less studied protein in comparison to Mpro. Three papers report on its inhibitors, including Pitsillou et al., who focus on two different aspects: 1) investigating the binding characteristics of previously identified, by a high-throughput screening, Naphthalene-based inhibitors against the SARS-CoV-2 papain-like proteases (PLpro) and 2) evaluating their effect on PLpro deubiquitinating activity. These findings are in accordance with the mechanisms and potential antiviral effects of the naphthalene-based, GRL-0617 inhibitor, which is currently progressing in preclinical trials. Their findings indicate further suitable candidates such as PLpro inhibitors, which are considered potential lead compounds.

Ibrahim et al. used three publicly docking tools, AutoDock Vina, PLANTS, and FRED against SARS-CoV-2 PLpro. In particular, the authors start from the assumption that SARS-CoV PLpro and SARS-CoV-2 PLpro share a 100% sequence identity for the binding site of small molecules. Moreover, they consider that the residues (Tyr269 and Gln270), important for recognition site, are present in both proteins, and generate a high-quality DEKOIS 2.0 benchmark set. In fact, since the co-crystal structure of SARS-CoV-2 PLpro with the commonly held small-molecule inhibitor is not reported, the authors make a homology model for SARS-CoV-2 PLpro complexed with a small-molecule ligand based on the ligand-bound SARS-CoV. FRED performed best against the built model, thus its screening performance and chemotype enrichment were comparable to the built model demonstrating the high quality of the built model. Therefore, they employed FRED in a VS campaign using the FDA-reported drugs (from DrugBank) against SARS-CoV-2 PLpro.

Huynh et al. investigated computational screening and the repurposing of FDA approved drugs through molecular modeling studies. The results of their study through MD simulations clarify that such compounds are not appropriate for the PLpro. Long range MD simulations suggest that the known inhibitor rac5c is bound stably inside the PLpro substrate binding pocket, and expose the molecular mechanism of the rac5c-PLpro complex. Detailed molecular level insights of rac5c are elucidated in the dynamic state by quoting the pyridine fragment (with attached -OCH3 group) loosely bound in the PLpro substrate binding pocket. From this, Huynh et al. suggested optimizing the loosely bound pyridine fragment with the alternative functional ground for the enhancement of binding affinity. Delre et al., have also analyzed the SARS-CoV-2 PLpro by repurposing the known compounds (688 phase III and 1,702 phase IV clinical trial drugs from the ChEMBL database) for Covalent and Non-covalent Inhibitors with desirable poly-pharmacology profiles. Delre et al. applied protein–ligand interaction fingerprint similarities, conventional docking scores, and MM-GBSA–binding free energies for executing the repurpose drugs through a covalent inhibition of PLpro.

Besides better known targets, there are also other equally important targets that can be used to fight the virus, as explored by several articles in this special issue. Yang et al. determined at a resolution of 2.0 Å the crystal structure of the SARS-CoV-2 nucleocapsid protein C-terminal domain (CTD). The CTD was shown to have a comparable distinct electrostatic potential surface to the equivalent domains of other reported CoVs, suggesting that the CTD has novel roles in viral RNA binding and transcriptional regulation. In particular, the crystal structure of the nucleocapsid CTD was analyzed, the potential self-interaction formation of SARS-CoV-2 N-CTD was studied, and the self-interaction characteristics of the single-point mutant were verified. By studying the recognition mechanism of SARS-CoV-2 N-CTD protein to viral genomic intergenic transcriptional regulatory sequences (TRSs), what emerges is that the nucleocapsid protein CTD is responsible for the discontinuous viral transcription mechanism by recognizing the different patterns of viral TRS during transcription and revealing a new method of viral transcription sequences mechanism.

El Hassab et al. employed Computer-aided drug design (CADD) techniques for the identification of a novel inhibitor for SARS-COV-2 RNA-dependent RNA polymerase. Such an approach indicates MAW-22 as a potential new inhibitor. MAW-22 demonstrated a strong binding affinity and energy profile for SARS-CoV-2 polymerase even better than the known antiviral drug remdesivir so to suggest that it could be used as an effective agent for the management of SARS-CoV-2 infection. Moreover, this study indicates that CADD is an efficient tool to develop drugs for treating SARS-CoV-2 infection. The aim of this study was not only to design a potential inhibitor but also to establish guidance for future drug development for COVID-19 infection.

Munaweera and Hu employed computational techniques to identify molecules that are able to inhibit SARS-CoV-2 nsp14 at the exoribonuclease (ExoN) site. Many nucleoside analogues (NuA) are known to lead to lethal mutagenesis for the viruses. The success of their activity can be made in vain by the proofreading activity of the ExoN. Thus, the simultaneous use of NuA and nsp14 inhibitors could enhance lethal mutagenesis in SARS-CoV-2. With this in mind, the authors built a homology model using the nsp14 of SARS-CoV as a template by molecular-docking, identified a potential lead molecule, PV6R, which belongs to DEDDh/DEEDh subfamily nuclease inhibitors, and can bind to the ExoN binding site of nsp14. Moreover, PV6R was computationally characterized and its molecular features were extracted and used to perform a virtual screening, by which different molecules were identified and successively optimized by computational strategies.

Squeglia et al. focused their attention on the host DEAD-box (DDX) RNA helicases, hijacked by coronaviruses to play key roles in viral replication steps. The highly conserved viral proteins responsible for DDX interactions probably use common pathways to exploit host proteins for their replication. In this review, the authors produce structural and functional data for considering DDXs as the possible key factors involved in SARS-CoV-2 hijacking mechanisms, exploring possible interactions between human DDX and coronavirus proteins, by integrating the available structural information with homology modeling studies. Furthermore, they hypothesize a double role related to the DDX helicases hijacking by coronaviruses: by enhancing key steps of the virus RNA replication/transcription and, simultaneously, by repressing the host innate immune response. Finally, DDX helicases could be considered novel targets for antiviral therapy also against SARS-CoV-2, as already validated for other RNA viruses.

Rampogu and Lee provided the structure-based pharmacophore modeling approaches for finding suitable inhibitors for SARS-CoV-2 2′- O -methyltransferase (nsp16/nsp10 complex) enzyme with FDA approved drug library compounds. The structure-based pharmacophore screening yields the three best compounds, which are better than the natural substrate S-adenosyl methionine (SAM), and inhibitors like remdesivir and hydroxychloroquine. From the pharmacophore based molecular docking and MD simulation approaches, Rampogu and Lee suggested that framycetin, kanamycin, and tobramycin could be strong and potential SARS-CoV-2 2′- O -methyltransferase inhibitors.

Žerovnik addresses a mini review on the Pore-forming proteins (PFPs) that virtually appear in all organisms and which cause ion dis-balance, small substances, or even protein efflux/influx, influencing a cell’s signaling routes and fate by disrupting cellular membranes, depending on the pore size. In particular, these proteins are considered possible ways for therapy of channelopathies and/or modulating immunity relevant to the new threat of SARS-CoV-2 infections. Žerovnik summarized the current knowledge regarding the comparative features and mechanisms of pore formation by amyloid-forming proteins (AFPs), anti-microbial peptides (AMPs) and viroporins, and transmembrane short viral envelope proteins (E protein), helping spread certain viruses, among them the coronavirus SARS-CoV-2. The paper stresses that finding common mechanisms could be useful to design common means of defense and augment anti-viral and anti-amyloid therapies.

This Research Topic on “*Molecular Studies of Covid-19 Chemistry*” provides an overview of current knowledge and highlights interesting new insights into recognizing new targets and identifying or repurposing compounds that can combat the virus.

